# An integrated lncRNA, microRNA and mRNA signature to improve prognosis prediction of colorectal cancer

**DOI:** 10.18632/oncotarget.20013

**Published:** 2017-08-07

**Authors:** Yongfu Xiong, Rong Wang, Linglong Peng, Wenxian You, Jinlai Wei, Shouru Zhang, Xingye Wu, Jinbao Guo, Jun Xu, Zhenbing Lv, Zhongxue Fu

**Affiliations:** ^1^ Department of Gastrointestinal Surgery, The First Affiliated Hospital of Chongqing Medical University, Chongqing 400016, China

**Keywords:** colon cancer, multi-RNA-based classifier, prognosis, TNM stage

## Abstract

Although the outcome of patients with colorectal cancer (CRC) has improved significantly, prognosis evaluation still presents challenges due to the disease heterogeneity. Increasing evidences revealed the close correlation between aberrant expression of certain RNAs and the prognosis. We envisioned that combined multiple types of RNAs into a single classifier could improve postoperative risk classification and add prognostic value to the current stage system. Firstly, differentially expressed RNAs including mRNAs, miRNAs and lncRNAs were identified by two different algorithms. Then survival and LASSO analysis was conducted to screen survival-related DERs and build a multi-RNA-based classifier for CRC patient stratification. The prognostic value of the classifier was self-validated in the TCGA CRC cohort and further validated in an external independent set. Finally, survival receiver operating characteristic analysis was used to assess the performance of prognostic prediction. We found that the multi-RNA-based classifier consisted by 12 mRNAs, 1miRNA and 1 lncRNA, which could divide the patients into high and low risk groups with significantly different overall survival (training set: HR 2.54, 95%CI 1.67-3.87, p<0.0001; internal testing set: HR 2.54, 95%CI 1.67-3.87, p<0.0001; validation set: HR 5.02, 95% CI 2.2–11.6; p=0·0002). In addition, the classifier is not only independent of clinical features but also with a similar prognostic ability to the well-established TNM stage (AUC of ROC 0.83 versus 0.74, 95% CI = 0.608-0.824, P =0.0878). Furthermore, combination of the multi-RNA-based classifier with clinical features was a more powerful predictor of prognosis than either of the two parameters alone. In conclusion, the multi-RNA-based classifier may have important clinical implications in the selection of patients with CRC who are at high risk of mortality and add prognostic value to the current stage system.

## INTRODUCTION

Despite advances in screening, diagnosis, and curative resection, colorectal cancer (CRC) is still the third most common epithelial malignancy and the fourth-leading cause of mortality around the world [1–3]. At present, the tumor, node, and metastasis (TNM) staging is the only well-recognized stratification system used in clinical practice to guide therapy decision and predict CRC patients’ prognosis [4]. However, TNM staging fails to evaluate survival outcomes accurately in many patients undergoing surgical resection [5]. Conflict outcomes even existed among patients with same stage category [6, 7]. Besides that, although the version of TNM staging has been continuously updating in the past decade, the prognosis value does not increase significantly [8]. All the above factors highlight the urgent need to identify reliable prognostic factors for a more precise prediction in CRC patients [9–11].

The accumulation of basic research revealed that certain molecules which intimately associated with tumor phenotype and clinical behavior, maght be with better predictive value than clinicopathological features [12]. Indeed, many previous studies had confirmed that the discovery and application of individual biomarkers could facilitate the prognostic evaluation [9–11]. But due to their specificity and sensitivity, individual molecular alone or in combination with clinical features also do not predict the survival of CRC patients accurately [13].

Given the heterogeneity of CRC and the multitude of variables influencing clinical progress, the multi-molecular signatures provide a more comprehensive prognostic information. Expression levels of thousands of molecular are now widely evaluated simultaneously by microarray, sequencing and mass spectrometry due to huge breakthrough of high-throughput technology during the last decade [14]. Therefore, expression profiling especially mRNA, miRNA and lncRNA, has shown great prospect in clinical practice to predict the long-term outcome of individual patient. Moreover, many researches have demonstrated that, notwithstanding the importance of individual RNA, intrinsic multi-RNA profiles of CRC may have greater prognostic value. Ramon Salazar et al. reported an expression profiling study and screened 18 mRNAs that could significantly improve the prognostic accuracy in patients with stage II and III CRC [15]. In another study, by integrating 7 genes into a single model, Anita Sveen et al constructed a prognostic classifier for stage III CRC patients and validated that the classifier is a clinically feasible prognostic predictor of poor prognosis [16]. Besides that, MicroRNAs (miRNAs) and long non-coding RNAs (lncRNAs) as key ingredients of expression profiling involved in directly regulating approximately 30% of protein-encoding genes [17]. Although functions of miRNAs are far from being fully understood, growing evidences indicated that aberrant expression of miRNAs meet the requirement of ideal biomarkers for cancer detection [18], which were not only stable in plasma and cuticle at detectable levels [19], but also showed a good sensitivity and specificity [20]. Thus, the prognostic value of individual miRNA in CRC was continuously being reported [21, 22] since it was first discovered by Lee in 1993 [23]. Similarly, recent investigations on various human cancers demonstrated that lncRNAs may be an overlooked source of biomarkers and therapeutic targets [24]. Although only a limited number of lncRNAs have been well characterized on biological mechanism, accumulating evidences have suggested that lncRNAs may have significant prognostic value in many types of cancers [25] including breast cancer [26], prostate cancer [27] and CRC [28, 29].

Despite much was known about RNAs in CRC, previous studies mainly focused on them separately. It is still unknown whether combining different types of RNAs could substantially increase the prognostic value. Therefore, the aims of this present work was to construct a multi-RNA-based classifier based on exploring the lncRNA, miRNA and mRNA profiles of CRC patients. The prognostic value of the classifier was investigated in training cohort and further confirmed in independent validation cohort. Our findings suggest that the multi-RNA-based classifier could effectively stratify CRC patients who are at high risk of mortality and add prognostic value to the current stage system.

## RESULTS

### Clinicopathological features of patients in the TCGA and validation CRC cohort

**Figure 1:**
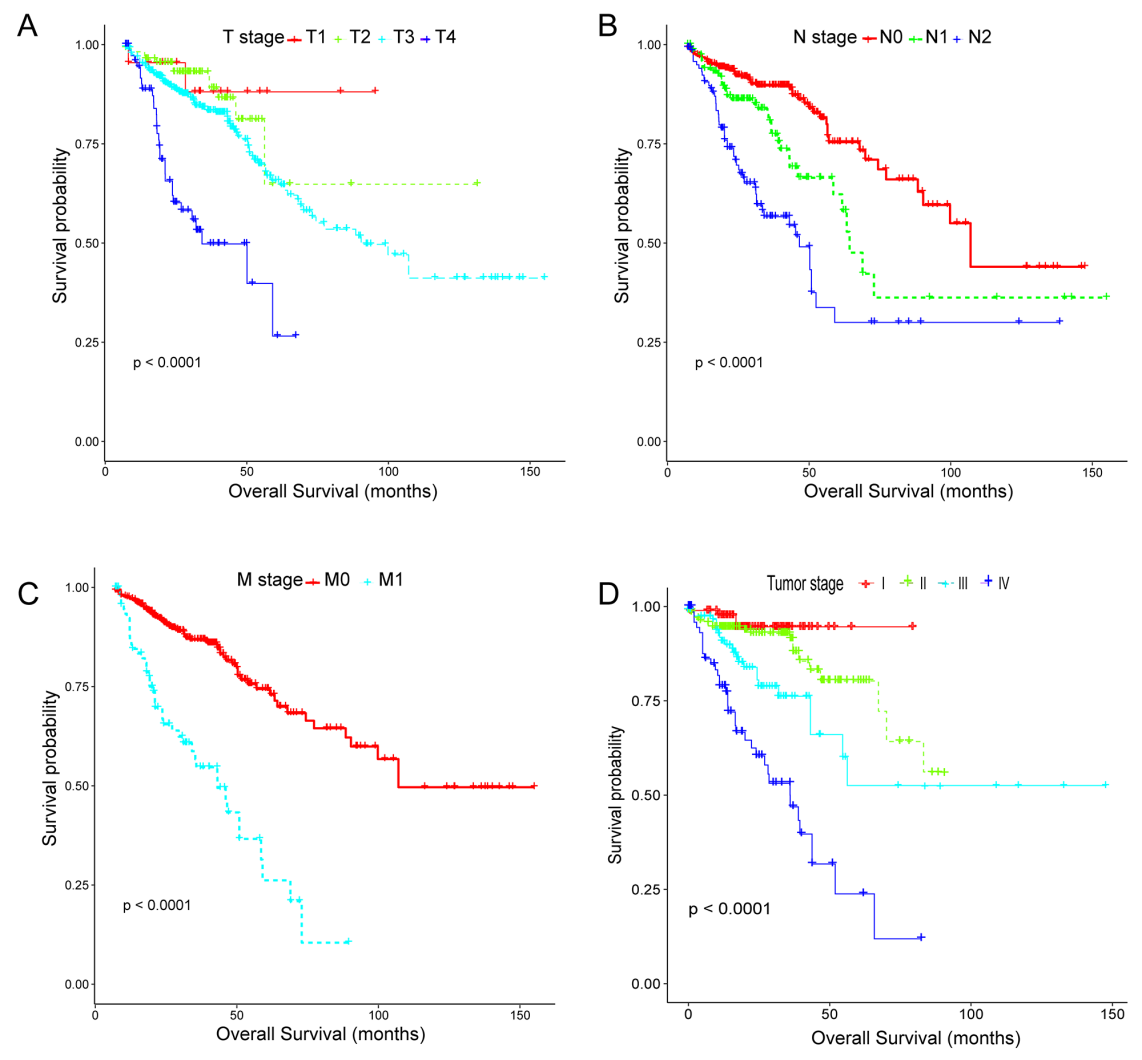
Kaplan-Meier estimates of overall survival stratified by the tumor, node, metastasis system and TNM stage in the TCGA CRC cohort **(A-C)** Local invasion stage (T1, T2, T3, T4), lymph node metastasis (N0, N1, N2), distant metastasis (M0, M1); overall log-rank test, p-value <0.0001. **(D)** TNM stage, overall log-rank test, p-value <0.0001. The differences between the two curves were determined by the two-sided log-rank test.

Two CRC cohort and corresponding clinical data were downloaded from the publicly available TCGA and GEO database, respectively. After removal of the samples with inadequate clinical information, a total of 663 CRC patients including 338 females (mean age 66.04 ± 13.76 years) and 325 males (mean age 66.63 ± 11.43 years), were analyzed in the present study (median follow-up: 23.98 months). All the included patients were pathologically diagnosed with CRC and undergone surgical resection, in which 598 patients from TCGA database were randomly divided into training set (n=498) and internal testing set (n=100) separately, and 65 patients from GEO database (GSE29623) possessing lncRNA, miRNA and mRNA profiles simultaneously were set as validation set [30]. Of note, 50 patients with expression profiles derived from adjacent non-tumor tissues were specifically assigned to training set for analyzing the differential expression of RNAs. Demographic and clinical data for the training, internal testing and independent validation set were summarized in Table 1. As we expected, no significant difference was observed in the major clinicopathological characteristics. However, owing to the high censoring rate in the TCGA CRC cohort(67.15%), remarkable difference existed in overall survival status(*p*<0·0001). Thus, Kaplan-Meier tests were subsequently conducted to evaluate the accuracy of the survival data. As clearly indicated in Figure 1, although containing a majority of censored data, the survival information in the TCGA CRC cohort was significantly related to the well-established TNM stage, which means its accuracy was appropriate for use in further studies.

**Table 1:** Clinical features for the CRC patients in the training set, testing set and validation set

Characteristics	TCGA cohort		Validation cohort	P-value^a^
	Training set n = 498(%)	Testing set n =100(%)	Independent set n = 65 (%)	
Age (years)				
< 60	144(28.9%)	28(28.0%)	20(30.8%)	0.8696
≥ 60	354(71.1%)	72(72.0%)	45(69.2%)	
Sex				
Female	255(51.2%)	52(52.0%)	31(47.6%)	0.1705
Male	243(48.8%)	48(48.0%)	34(52.4%)	
Local invasion				
T1–T2	107(21.4%)	21(21.0%)	13(20.0%)	0.9091
T3–T4	391(78.6%)	79(79.0%)	52(80.0%)	
Lymph node metastasis				
N0	303(60.8%)	61(61.0%)	38(58.4%)	0.1583
N1	108(21.6%)	20(20.0%)	20(30.7%)	
N2	87(17.6%)	19(19.0%)	7(10.9%)	
Distant metastasis				
M0	419(84.1%)	81(81.0%)	47(72.3%)	0.04777
M1	79(15.9%)	14(14.0%)	18(27.7%)	
TNM stage				
I	96(19.2%)	19(19.0%)	7(10.8%)	0.0514
II	197(39.5%)	41(41.0%)	22(34.0%)	
III	126(25.3%)	26(26.0%)	18(27.6%)	
IV	79(16.0%)	14(14.0%)	18(27.6%)	
Resection Margin Status				
R0	454(91.1%)	91(91.0%)	57(87.6%)	0.05582
R1	5(1.0%)	11(11.0%)	3(3.0%)	
R2	39(7.9%)	88(88.0%)	7(7.6%)	
Tumor grade				
Well	398(81.3%)	79(79.0%)	51(79.6%)	0.8549
Mod	65(13.1%)	14(14.0%)	10(15.3%)	
Poorly	35(5.6%)	77(77.0%)	4(5.1%)	
Relapse status				
No	377(75.7%)	73(73.0%)	51(78.4%)	0.8549
Yes	121(24.3%)	27(27.0%)	12(21.6%)	
Survival status				
No	413(83.0%)	79(79.0%)	40(61.5%)	< 0.0001
Yes	85 (17.0%)	21(21.0%)	25(38.5%)	

^a^Pearson chi-square test or Fisher exact test was used for comparison between subgroups. Bold type indicates statistical significance.

### Construction of prognostic classifier from the training sets

**Figure 2:**
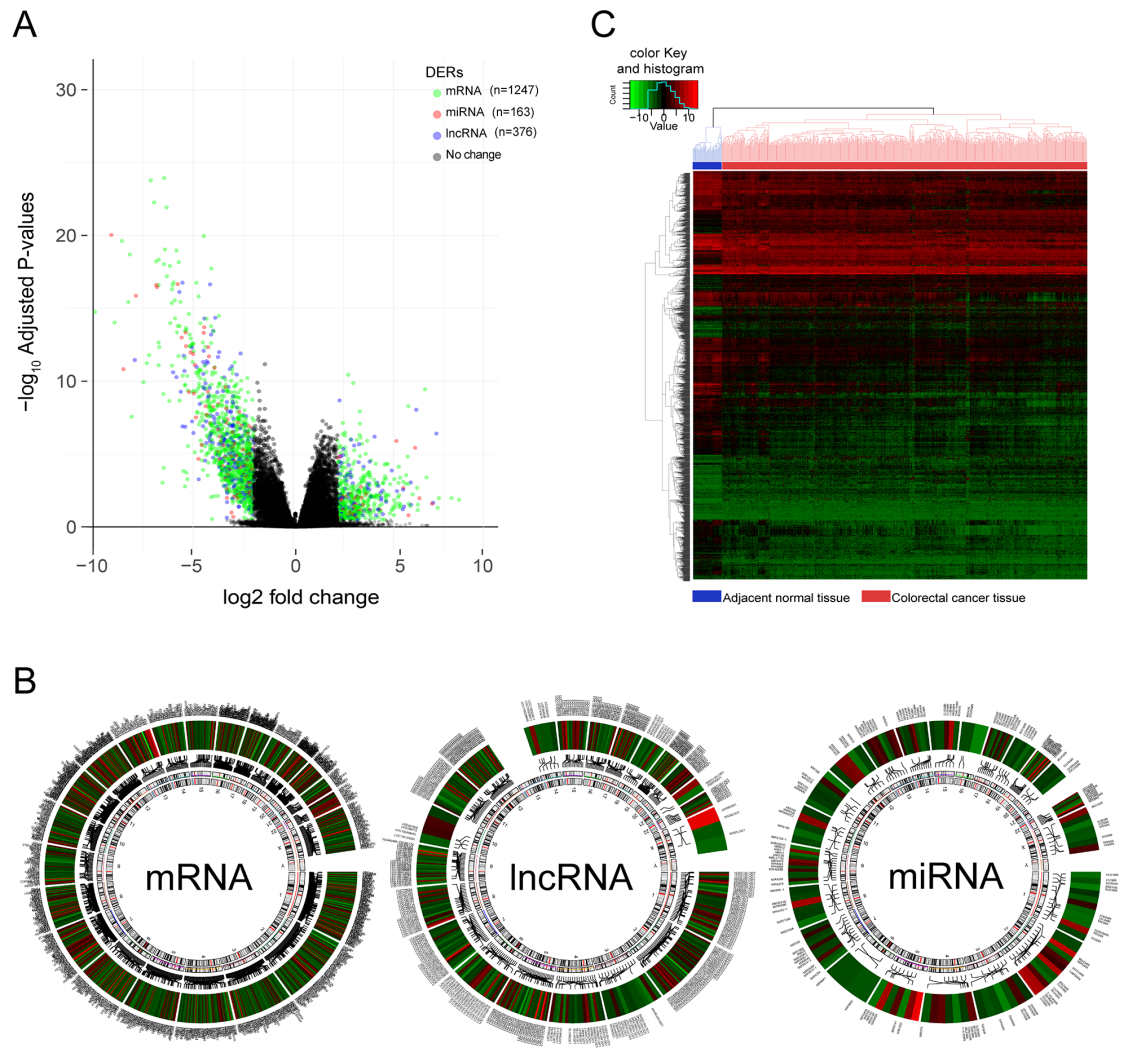
Differentially expressed RNAs(DERs) in colorectal cancer vs adjacent normal tissues **(A)** Volcano Plot visualizing the DERs which was screened by both limma and edgeR. The colorized points in scatter plot represent the DERs with statistical significance (adj.P.Value<0.05, |logFC|>2). Green, red and blue point represent mRNA, miRNA, lncRNA respectively. **(B)** The distribution and variation trend of each DERs on chromosomes was shown in Circos plots. Color gradient from red to blue represent the logFC of DERs, the gene symbol of each DERs was displayed in outermost region and been pointed to a specific location on chromosome by a connecting line. **(C)** Two-way hierarchical clustering of 498 tumour tissues and 50 adjacent normal mucosa with the 2114 differentially expressed RNAs using Euclidean distance and average linkage clustering. Every row represents an individual gene, and each column represents an individual sample. color gradient from green to red indicate expression levels from low to high on a normalized value(-10 to 10). The clustering of samples were mainly divided into two major clusters, one was the normal tissue samples and the other was cancer tissue samples.

**Figure 3:**
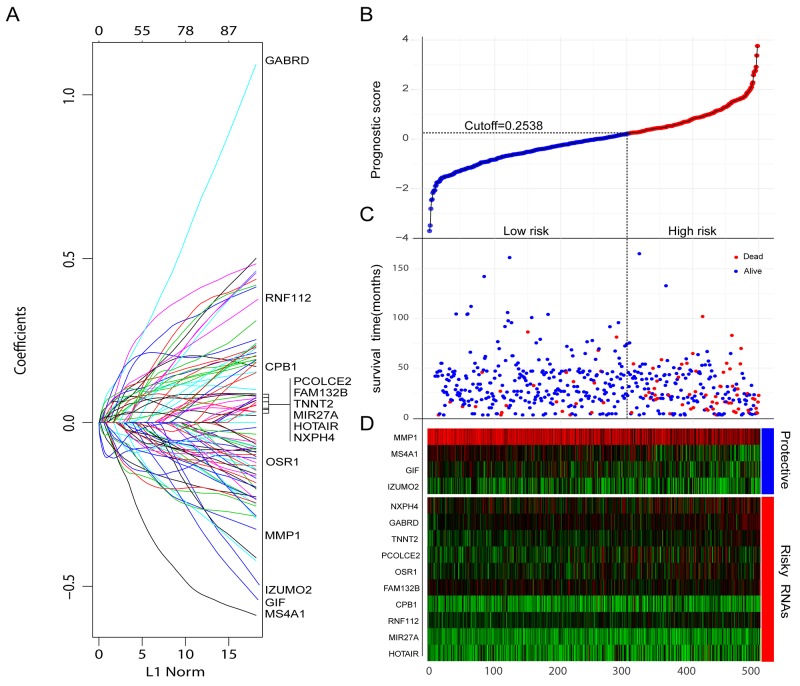
Construction of the integrated prognostic classifier **(A)** LASSO coefficient profiles of the 14 survival-related RNAs. **(B-D)** Prognostic classifier analysis of the training set patients. **(B)** The distribution of risk score. **(C)** Patients survival time and status. The black dotted line represents the optimum cutoff dividing patients into low-risk and high-risk groups. **(D)** Heat map of the RNAs in prognostic classifier.

Aberrant expression of RNAs which mediated tumor initiation, progression, and metastasis is the potential prognostic biomarker. To obtain robust and reproducible differentially expressed RNAs (DERs), global expression profiling was conducted on TCGA training set including 498 CRC specimens and 50 adjacent normal tissues, by using redgeR and limma respectively. A total of 1786 DERs were preliminarily screened by both the algorithms with the threshold of |log2FC| >2 and adj.*P*.Value < 0.05, in which the number of mRNA, miRNA and lncRNA was 1247, 163, 376 respectively (Figure 2A). The details of DERs was comprehensively displayed in circos plot (Figure 2B). Moreover, as shown in Figure 2C, using unsupervised hierarchical clustering based on those DERs, the samples of tumor and normal were clearly separated into two discrete groups which indicated that the DERs identified in the present study were credible. Based on univariate survival analysis, the DERs in which not significantly correlated with overall survival was further filtered out. Then, a relative regression coefficient was calculated by LASSO analysis based on the survival-related DERs. By forcing the sum of the absolute value of the regression coefficients to be less than a fixed value, certain coefficients were shrunk exactly to zero and the most powerful prognostic marker of all the CRC survival-associated DERs were selected including 12 mRNAs, 1miRNA and 1 lncRNA (Figure 2A). As summarized in Table 2 , ten were associated with high risk (FAM132B, CPB1, NXPH4, OSR1, PCOLCE2, RNF112, TNNT2, GABRD, MIR27A, HOTAIR, HR>1) and four were shown to be protective (MMP1, MS4A1, IZUMO2, GIF, HR<1). Combine the relative expression levels of the DERs in the classifier and corresponding LASSO coefficients, a risk score (RS) was calculated for each patient in the TCGA training set. The cutoff point of RS for dividing the high-risk and low-risk patients was generated according to the optimum sensitivity (74.27%) and specificity (84.68%) based on ROC curve for predicting 5-year survival. Patients with a RS greater than or equal to 0.2835 were assigned to high-risk group and the rest patients belonged to low-risk group (Figure 3B). As shown in Figure 3C and 3D, we found that patients with high RS tended to express high-risk RNAs, whereas tumors with low RS incline to express protective RNAs.

**Table 2:** The RNA in the multi-RNA-based classifier significantly associated with survival of CRC patients in training set

Symbol	Chromosome location	Type	Univariate Cox regression analysis			LASSO coefficient
			HR	95% CI	P value	
Protective RNAs						
MMP1	11q22.3	mRNA	0.854	0.784-0.932	0.001	-0.068643
MS4A1	11q12	mRNA	0.869	0.799-0.945	0.001	-0.030945
IZUMO2	19q13.33	mRNA	0.897	0.822-0.945	0.014	-0.024213
GIF	11q13	mRNA	0.881	0.806-0.961	0.005	-0.014259
Risky RNAs						
FAM132B	2q37.3	mRNA	1.140	1.001-1.297	0.048	0.003712
CPB1	3q24	mRNA	1.110	1.019-1.207	0.016	0.007147
NXPH4	12q13.3	mRNA	1.108	1.023-1.200	0.012	0.008292
OSR1	2p24.1	mRNA	1.172	1.048-1.312	0.005	0.014616
PCOLCE2	3q21-q24	mRNA	1.135	1.038-1.240	0.005	0.025477
RNF112	17p11.2	mRNA	1.276	1.082-1.495	0.003	0.035002
TNNT2	1q32	mRNA	1.236	1.089-1.410	0.002	0.045565
GABRD	1p36.3	mRNA	1.315	1.096-1.577	0.003	0.046474
MIR27A	19p13.13	miRNA	1.191	1.035-1.370	0.015	0.016724
HOTAIR	12q13.13	lncRNA	1.112	1.027-1.203	0.008	0.004548

HR, hazard ratio; CI, confidential interval.

### Validation of the integrated classifier for survival prediction in the TCGA and validation CRC cohort

**Figure 4:**
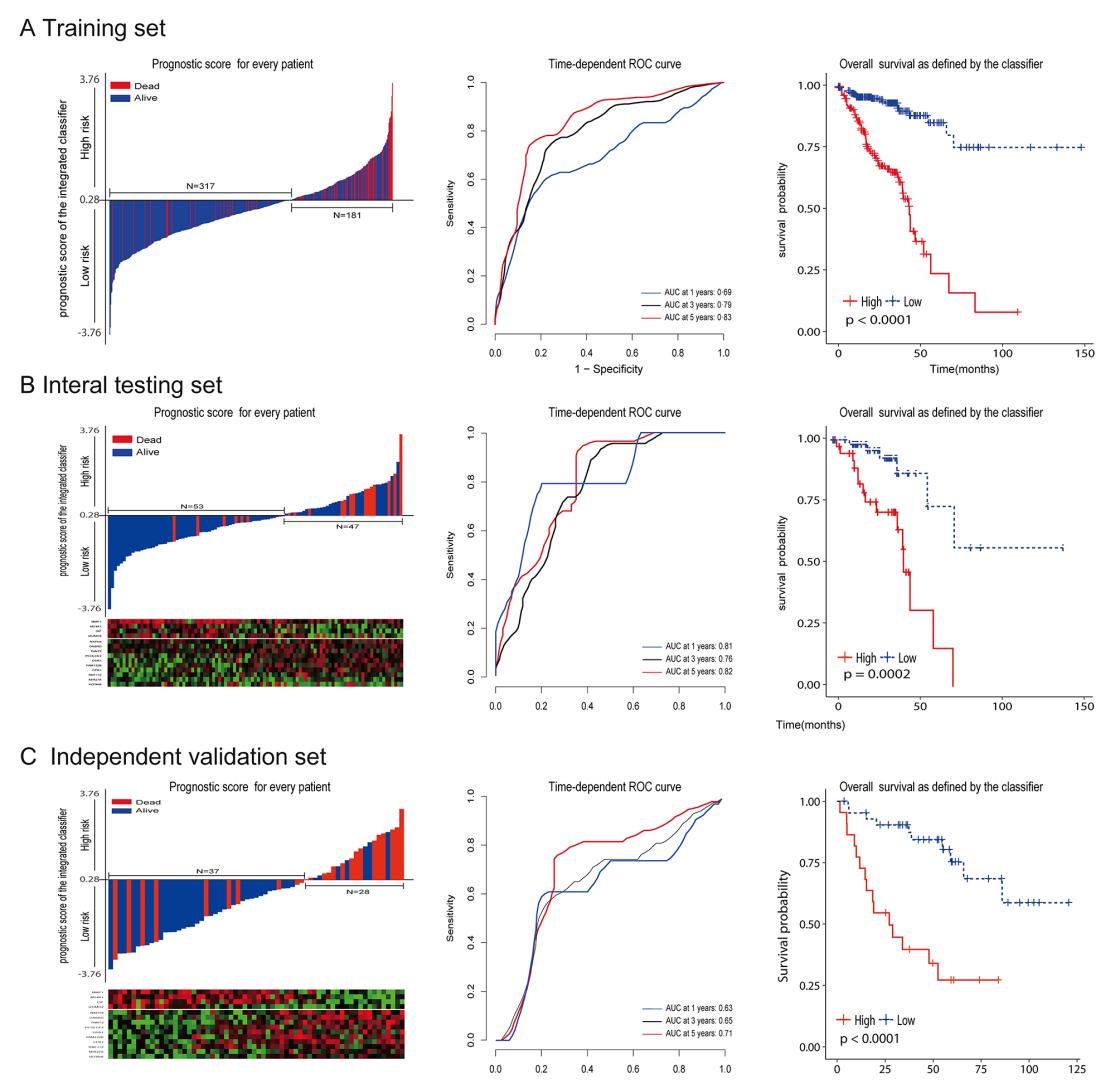
The distribution of risk score, time-dependent ROC curves and Kaplan-Meier survival based on the integrated classifier in the training, internal testing, and independent validation sets ROC, receiver operator characteristic. AUC, area under the curve. **(A)** Training cohort. **(B)** Internal testing cohort. **(C)** Independent validation cohort. We used AUCs at 1, 3, and 5 years to assess prognostic accuracy, and calculated *p* values using the log-rank test.

To investigate the relationship between RS and survival status of CRC patients, Kaplan-Meier analysis and log-rank test were conducted on the training sets. Obviously, patients with higher RS generally had a significantly worse overall survival (OS) than those with lower RS (*p*<0.0001; Figure 4A ). As the majority of events occurred within 5 years, time-dependent ROC curves were used to assess the prognostic power based on OS at 1, 3, 5 years respectively (Figure 4A). The cumulative 5-year OS rate was 79.7% (95% CI 68.4–92.8) for the low-risk group, whereas it was only 15.7% (95% CI 5.2–47.1) for the high-risk group (hazard ratio [HR] 5.63, 95%CI 3.44–9.24; *p*<0·0001; Figure 2A). In addition, we did the same analyses on the internal testing set. As shown in Figure 4B, the results were similar to what we observed in the training set (HR 2.54, 95%CI 1.67-3.87; *p*<0.0001).

To validate whether our prognostic classifier also had similar predictive ability in different populations, we applied it to the independent set. There were only a limited number of patients (N=65) in the independent set because of lack of published datasets that have the mRNA, miRNA and lncRNA profiles for the same CRC patients. However, the variables (N= 15) to sample size (n=65) ratio was enough to accurately estimate the regression coefficients based on ridge regression. By using the same prognostic model and cutoff value (RS = 0.2835), the classifier can also successfully subdivide patients into high risk (n = 28) or low risk (n = 37) groups with remarkable differences in overall survival (HR 5.02, 95% CI 2.2–11.6; *p*=0·0002; Figure 4C). In consistence with the results demonstrated above, 5-year OS was 68.4% (95% CI 51.6–90.6) for the low-risk group, whereas it was only 27.2% (95% CI 12.8 –57.4 ) for the high-risk group.

### Prognostic value of the integrated classifier is independent of clinical feature

**Figure 5:**
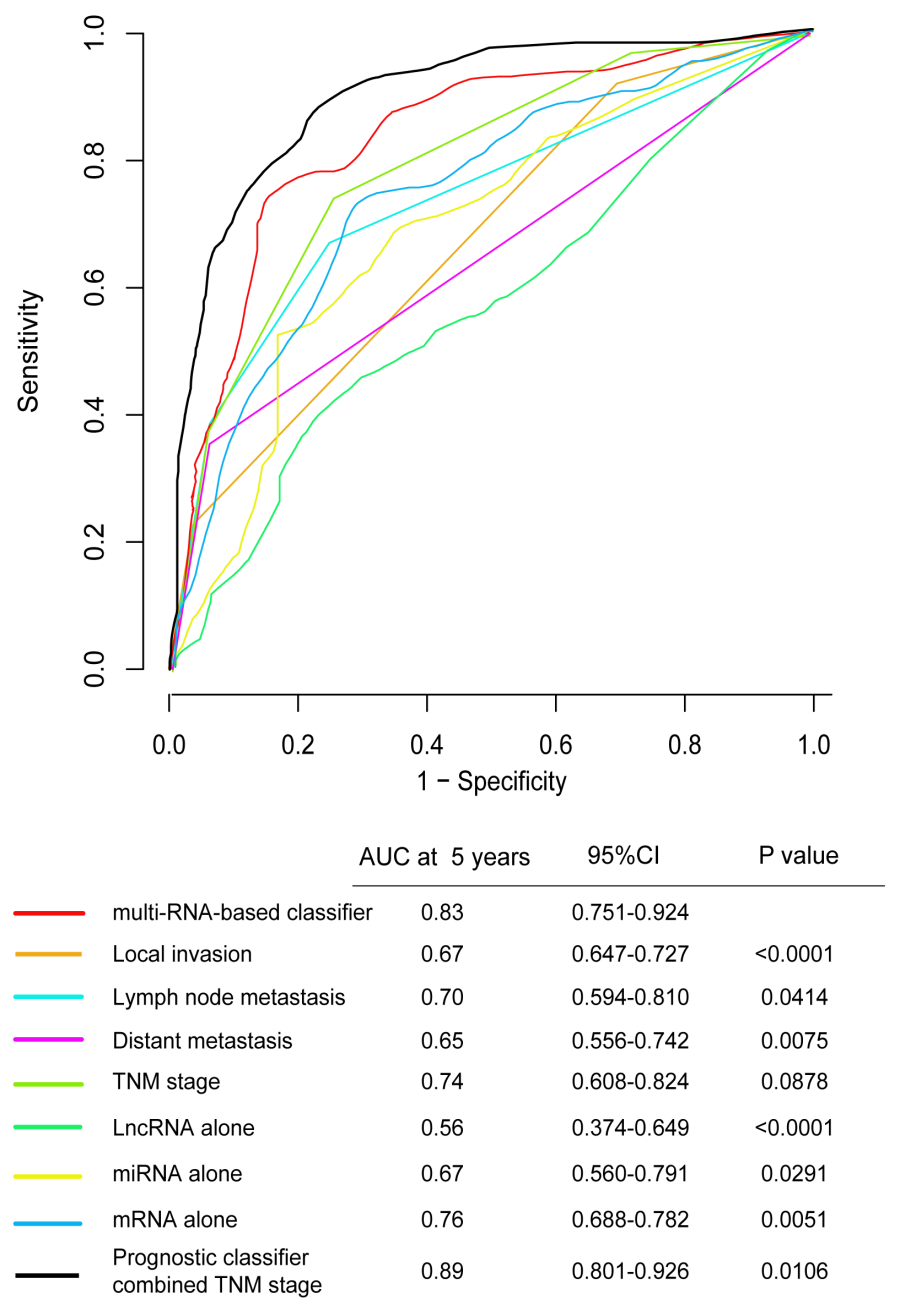
Time-dependent ROC curves compare the prognostic accuracy among the prognostic classifier, clinicopathological features and mRNA, miRNA, lncRNA alone in 598 patients Local invasion(T1-T4), lymph node metastasis(N0-N2), distant metastasis(M0-M1), TNM stage(I-IV), prognostic Score(risk score). Only lncRNA (HOTAIR), only mRNA (FAM132B, CPB1, NXPH4, OSR1, PCOLCE2, RNF112, TNNT2, GABRD, MMP1, MS4A1, IZUMO2, GIF), only miRNA (MIR27A). The 95%CI of AUC were calculated from 1000 bootstrap of the survival data. p values show the AUC at 5 years for multi-RNA-based classifier vs the AUC at 5 years for other features. ROC, receiver operator characteristic. AUC, area under curve.

**Figure 6:**
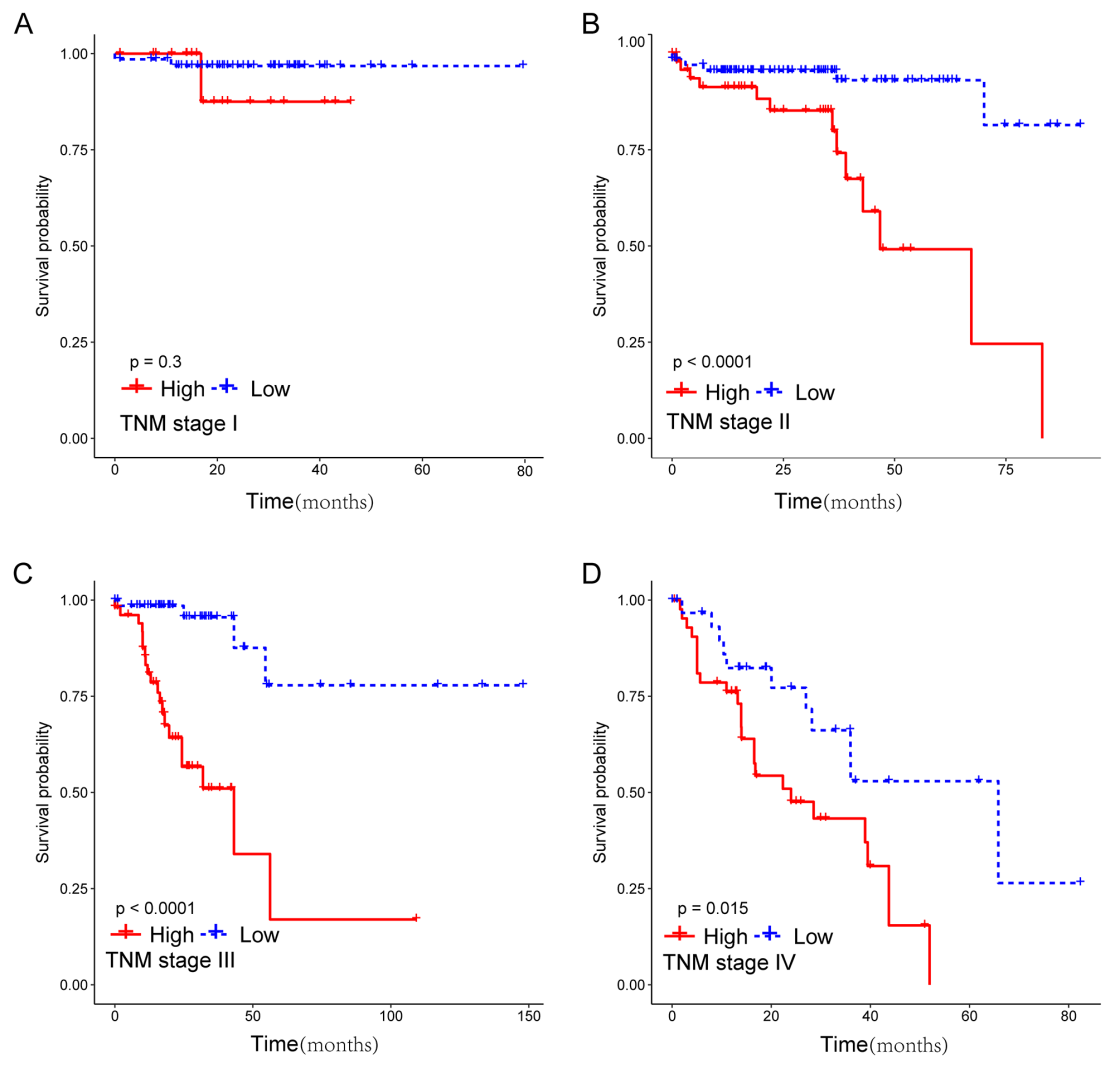
Kaplan-Meier survival analysis for 633 patients according to the prognostic classifier stratified by TNM stage Patients were stratified based on TNM stage (stage I, stage II, stage III and stage IV), and then Kaplan–Meier plots were used to visualize the survival probabilities for the low-risk versus high-risk group. **(A)** Kaplan–Meier curves for stage I (N=122); **(B)** Kaplan–Meier curves for stage II (N=250); **(C)** Kaplan–Meier curves for stage III (N=162); **(D)** Kaplan–Meier curves for stage IV (N=99).

**Figure 7:**
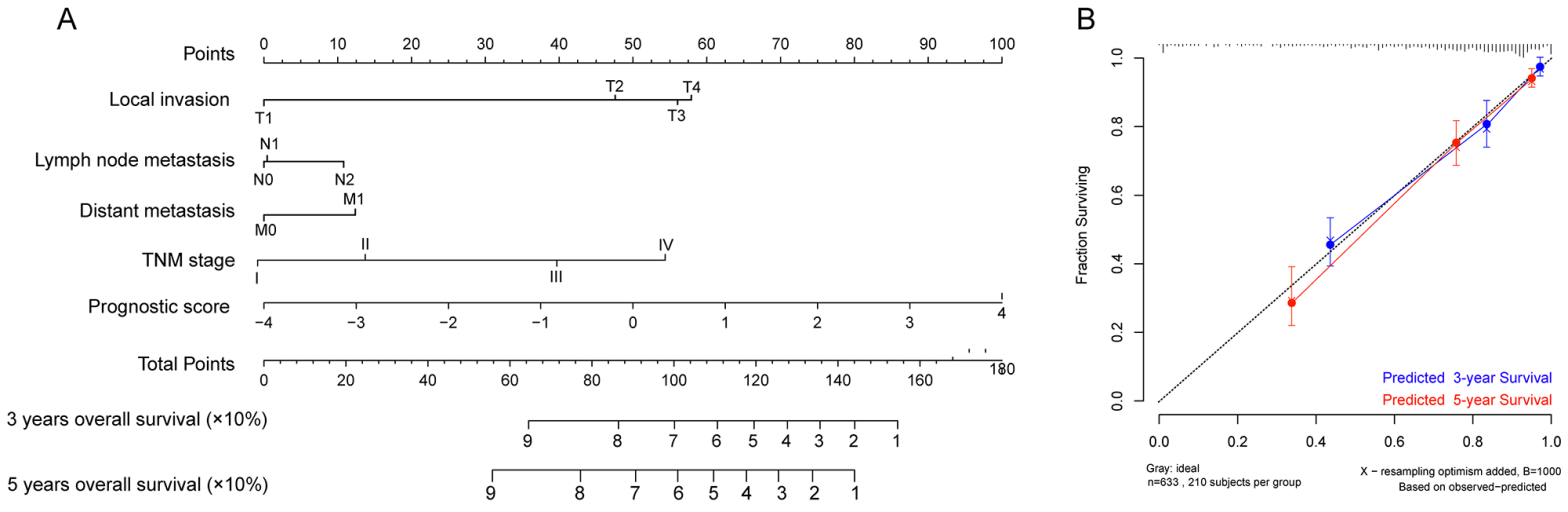
Nomograms to predict 3-year and 5-year survival probability in colorectal cancer **(A)** Total points were obtained by adding up the corresponding points of each individual covariate on the points scale. Then directly convert total points to particular 3–year and 5–year related survival probabilities. **(B)** Calibration plot for the nomogram. Dashed line indicates ideal reference line where predicted probabilities would match the observed proportions. Dashes represent nomogram-predicted probabilities grouped for each of the there groups, along with the respective confidence Intervals.

To assess whether the prognostic classifier represents an independent indicator in CRC patients, the effect of each clinicopathologic feature on survival was analyzed by Cox regression. As shown in Table 3 , after multivariable adjustment, the multi-RNA-based classifier remained a powerful and independent factor in training set, testing set and independent set. Additionally, time-dependent receiver operating characteristic (ROC) was applied to compare the predictive accuracy between the multi-RNA-based classifier and the other independent clinical factors (Figure 5). By calculating the area under the curve (AUC) of ROC, we found that the multi-RNA-based classifier had significantly higher prognostic accuracy than any other factors except TNM stage (0.83 versus 0.74, 95% CI = 0.608–0.824, P =0.0878). Although TNM stage is a well-recognized prediction system for prognosis, conflict outcome existed among patients with same stage category. To investigate whether the multi-RNA-based classifier adds prognostic value to the current system, data stratification analysis was performed. As shown in Figure 6, within each stratum (stage II-IV), our classifier could further subdivide the patients into longer survival and shorter survival group (Figure 6B–6D). More importantly, the multi-RNA-based classifier combined with clinical features achieved the greatest area under the curve of ROC which was significantly greater than classifier alone (0.89 versus 0.83, 95% CI 0.801–0.926, *P*=0.0106). These results demonstrated that combining the multi-RNA-based classifier with clinical features could further improve the capacity for predicting outcome. Therefore, we constructed a nomogram that integrated both the classifier and clinicopathological features to predict survival probability of patients who had undergone surgical resection (Figure 7A). Calibration plot showed that predicting 3-year and 5-year survival probabilities corresponded closely to the actual observed proportions (Figure 7B).

**Table 3:** Univariate and multivariate analyses of prognostic factors and overall survival of patients in the study

Variables	Categories	Univariate analysis		Multivariate analysis	
		HR (95% CI)	P value	HR (95% CI)	P value
**Training set, n=498**					
Age	≧60/<60years	1.263 (0.767-2.081)	0.358		
Sex	male/female	1.177 (0.767-2.081)	0.465		
Local invasion	T3-T4/T1-T2	3.798 (1.752-13.14)	0.013	2.206 (1.139-7.029)	0.027
Lymph node metastasis	N0/N1-N2	3.207 (2.026-5.075)	<0.0001	1.172 (0.672-2.044)	0.576
Distant metastasis	M0/M1	3.289 (3.397-7.235)	<0.0001	2.464 (1.363-4.453)	0.003
TNM stage	I-II/III-IV	4.348 (2.661-7.104)	<0.0001	2.151 (1.165-3.971)	0.014
Risk score(RS)	High/low	5.641 (3.441-9.245)	<0.0001	4.463 (2.687-7.413)	<0.0001
**Testing set, n=100**					
Age	≧60/<60years	1.126 (0.972-1.305)	0.112		
Sex	male/female	0.948 (0.864-1.041)	0.271		
Local invasion	T3-T4/T1-T2	2.008 (1.129-3.571)	0.017	1.503 (0.8583-2.634)	0.153
Lymph node metastasis	N0/N1-N2	2.729 (1.655-4.501)	0.0001	2.928 (0.197-3.933)	0.478
Distant metastasis	M0/M1	2.383 (1.906-2.978)	<0.0001	2.951 (1.122-7.759)	0.028
TNM stage	I-II/III-IV	3.177 (1.995-5.061)	<0.0001	3.548 (1.037-12.13)	0.009
Risk score(RS)	High/low	2.542 (1.671-3.879)	<0.0001	2.206 (1.139-7.029)	0.004
**Independent set, n=65**					
Age	≧60/<60years	1.247 (0.446-2.318)	0.612		
Sex	male/female	1.642 (0.845–3.120)	0.385		
Local invasion	T3-T4/T1-T2	1.677 (0.394-7.141)	0.483		
Lymph node metastasis	N0/N1-N2	2.969(1.123-7.845)	0.028	2.281 (1.189-3.784)	0.021
Distant metastasis	M0/M1	3.289 (3.397-7.235)	<0.0001	2.367 (1.394-4.019)	0.001
TNM stage	I-II/III-IV	5.008 (2.242-11.410)	<0.0001	2.138 (1.047-3.841)	0.025
Risk score(RS)	High/low	5.025 (2.259–11.643)	0.0002	4.247 (2.132-8.463)	<0.0001

HR, hazard ratio; CI, confidential interval.

## DISCUSSION

The increasing evidences are improving the understanding that, despite the importance of individual molecules, tumorigenesis and prognosis is strictly controlled by the interactions between the myriad of cellular constituents including DNA, RNA, proteins and small molecules [31]. Even a discrete biological function can rarely be ascribed to an individual molecule [32]. Therefore, based on an oversimplified model, which has dominated medical research for the past century, no longer applies to the current situation of data explosion in medical. The breakthrough of high-throughput technology was now powerful enough to measure each component of transcript in the tissue or cell at any given time [33, 34]. According to the estimate, the amount of bioinformation in the world doubles every 20 month, which promises to promote understanding of disease at the integration level and add new dimensions to our ability to predict the prognosis of an individual patient [35].

As being recognized previously, ribonucleic acid (RNA) is a polymeric molecule essential in various biological roles and many of which have been identified as prognostic biomarkers [18, 25]. Given the tumor heterogeneity and the multitude of variables involved in influencing clinical progress, combination of multiple RNA provides a more comprehensive prognostic information. Indeed, many former researches have revealed the great prospect in clinical utility of multi-RNA-based classifiers [36–38]. For example, using miRNA microarrays, Zhang et al. analysed 40 paired colon tumor and adjacent normal tissues and found that a six-miRNA-based classifier could predict disease recurrence and serve as an indicator of efficacy for adjuvant chemotherapy [39]. More importantly, the six-miRNA-based classifier even had better prognostic value than TNM stage and mismatch repair status. Additionally, it has been clarified recently that lncRNA is another dimension of transcription-regulatory networks [25, 37, 40, 41]. Aberrant expression of lncRNA is associated with tumorigenesis, tumor progression and metastasis [41]. Until now, although only a few lncRNAs have been investigated in CRC, existing results demonstrated that lncRNAs may be ideal prognostic biomarkers [25]. Thus, Hu et al. performed lncRNA expression profiling in large CRC cohorts from GEO and established a set of six lncRNAs that may be an efficacy tool for clinical prognosis evaluation [28]. Duo to the great power of multi-RNA-based risk stratification, its clinical utility has recently been approved by the FDA for making treatment decisions in early-stage breast cancer(MammaPrint; Agendia, Amsterdam, the Netherlands) [42, 43].

Although much is known about RNAs in CRC, previous studies mainly focused on them individually. The prognostic value of combining different type of RNAs is still not elucidated. More importantly, as the third most common epithelial malignancy, prognosis evaluation of patients with CRC based on current prognostic system still presents challenges [8]. Therefore, in the present study, we first constructed a novel multi-RNA-based classifier consisting of 12 mRNA, 1 miRNA and 1 lncRNA, which was validated as an independent predictor for CRC patient survival. Our data revealed that this classifier can successfully subdivide CRC patients into high and low risk groups with remarkable differences in overall survival. The results was further validated by a internal set and an independent external set, which reflects the good reproducibility of the classifier. In addition, even stratified by TNM stage, the CRC patients could also be divided into longer survival and shorter survival group by the multi-RNA-based classifier. And this further indicated that our classifier could act as an independent factor for CRC prognosis. More importantly, it is well known that CRC prognosis is highly stage dependent, however, dilemmas still exist in making appropriate treatment decisions, especially for a stage II patient. Therefore, identification of high-risk subgroup among stage II CRC patients by a reliable indicator is of great clinical need. Our data demonstrated that the multi-RNA-based classifier could be a promising biomarker to stratify stage II patients into distinct risk subgroup and guide individualized therapy choices.

Moreover, it was fascinating to find that the multi-RNA-based classifier and TNM stage had a similar prognostic ability and were independent of each other, which means the classifier may be used to refine the current prognostic model and facilitate further stratification of patients with CRC in the same TNM stage. Indeed, our ROC analysis indicated that a stronger power for prognostic prediction could been achieved by a combination of the multi-RNA-based classifier and clinicopathologic risk factors, which at least in part confirmed above conclusion. Intuition tells us that integrating different types of survival-related RNAs into a single model, instead of study on them separately, was expected to increase prognostic value substantially. Comparing the AUC of the ROC curve, we clearly found that removing any RNA type would significantly decrease the predictive ability. Therefore, single selective type of RNA was difficult to construct an sufficient precise prognosis model.

In this study, we evaluated the correction between survival and all the DERs, only CRC survival-related DERs were chosen to further analysis in LASSO algorithm. Finally, in our multi-RNA-based classifier, only 12 mRNA, 1 miRNA and 1 lncRNA were retained. Among them, MIR27A [44, 45], HOTAIR [46], MMP1 [47], MS4A1 [48], GIF [49] were previously reported to be related with CRC patient prognosis. As the only miRNAs, MIR27A directly downregulated the tumor suppressor FBXW7 and mediate selective activation of NOTCH, JUN and MYC signaling [45]. Moreover, long non-coding RNA HOTAIR in our classifier also silenced metastasis suppressor genes by recruiting the PRC2 complex to specific target genes [50]. These basic findings may, in part, account for the risky role of the two RNAs in classifier(HR > 1). Moreover, to our knowledge, we are the first to report the prognostic value of the other 9 RNAs(FAM132B, CPB1, NXPH4, OSR1, RNF112, TNNT2, IZUMO2, OSR1, PCOLCE2), which may provide valuable directions for the future research.

In summary, we constructed a powerful multi-RNA-based classifier which could effectively stratify CRC patients into groups at low and high risk of mortality. Further analysis revealed that our classifier was not only independent of clinical features but also with a similar prognostic ability to the well established TNM stage. Furthermore, to help clinician to evaluate survival probability of CRC patients, we integrated the multi-RNA-based classifier with traditional clinicopathological risk factors to make a quantitative nomogram. Base on our knowledge, this is the first report that combines multiple type of RNA to improve the current CRC prognosis. However, there were still some limitations in this research. In particular, some clinical information was incomplete, which made our study susceptible to the inherent biases. Moreover, the censoring rate of TCGA dataset was too high , which may affecte the reliability of this study. Clearly, our results should be further validated by prospective study in multicentre clinical trials.

## MATERIALS AND METHODS

### Data collection

All 635 patients’ data of CRC including RNA expression (mRNA, miRNA and lncRNA) and corresponding clinical information were retrieved from The Cancer Genome Atlas (TCGA) data portal. Both the expression profiles and clinical outcome are publicly available and open-access. Among the TCGA CRC cohort, 50 patients have expression data from both normal and tumor tissue simultaneously were used to screen differentially expressed RNAs (DERs). To validate the prognostic value of the integrated classifier obtained from the TCGA CRC cohort, external published datasets which have mRNA, lncRNA and miRNA profiles for the same CRC patients were retrieved from Gene Expression Omnibus(GEO). Finally, independent expression datasets with a total of 65 CRC patients were downloaded. Owing to the data were separately stored in different files, the barcodes of each sample were used to cross-reference the expression profiles and clinical outcome. The data collection was conducted in compliance with the publication guidelines and policies for the protection of human subjects provided by TCGA and GEO.

Before analyze the downloaded data, mRNA and miRNA expression profiles were annotated based on Refseq transcript ID and/ or Ensembl gene ID as previously described [2]. In addition, LncRNA expression profiles from patients in TCGA were retrieved from the Atlas of Non-coding RNAs in Cancer (TANRIC, http://bioinformatics.mdanderson.org/main/TANRIC:Overview) database [51], and any lncRNAs that overlapped with any given mRNAs were filtered out. By analysis of the download data, some patients do not meet the following criteria were eliminated in the present study: (1) a histological diagnosis of CRC (2) patients with fully clinical features including sex, age, tumor location, local invasion, lymph node metastasis, distal metastasis, differentiation grade, pathologic stage, survival information (Table 1); (3) patients were still alive at least 1 month after initial pathologic diagnosis.

### Identification of differentially expressed RNAs between CRC and normal tissue

The analysis was carried out in training set which contain 50 adjacent normal and 498 CRC sample, by using the R/Bioconductor package of redgeR and limma respectively, as previous described in detail. The expression differences were characterized by logFC (log2 fold change) and associated adj.*P*.Value. LogFC indicates the fold change in expression of each miRNA from CRC to normal tissue. Down and up-regulated RNAs were assigned a logFC < −2 and logFC >2 respectively, with adj.*P*.Value < 0.05. The RNAs identified to be differentially expressed by both of the algorithms were selected as DERs. In order to further assess the accurate of the DERs, hierarchical clustering analysis was also applied to categorize the data based on the similar expression patterns by using heatmap.2 function of the R/package gplots with complete linkage.

### Survival analysis

The differences clinical features including sex, age, tumor location, local invasion, lymph node metastasis, distal metastasis, differentiation grade, pathologic stage, survival status between training set, internal testing set and independent validation set were analyzed using the chi-square test. A univariate Cox model was performed to investigate the relationship between the continuous expression level of each DERs and OS, and for preliminary screening of clinical variables that were correlated with the OS of patients with CRC. Hazard ratios (HRs) and P-value from univariate Cox regression analysis were used to identify candidate survival-related DERs. DERs with HR for death > 1 were defined as high-risk RNAs a and those with HR < 1 were defined as a protective RNAs. The common DERs meet criteria of P-value <0.05 were selected as survival-related DERs and further analyzed in LASSO regression to identity the most powerful prognostic markers. Finally, a multi-RNA-based classifier was constructed for predicting the OS. In order to quantify the risk of OS, a standard form of risk score(RS) for each CRC patient was calculated combine the relative expression levels of the RNAs (*Exp*_i_) and LASSO coefficients (*L*_*i*_), $\text{Risk score}\;=\;\sum_{i=1}^{n}Exp_{i}\times L_{i}$. To divide the patients into the high or low risk group, the best cutoff RS was determined when the sensitivity and specificity in the ROC curve achieved optimum for predicting 5-year survival of the training set. Kaplan-Meier curves were used to estimate the survival for patients in training, testing and validation set between high risk and low risk group. As a powerful predictive factor, whether the prognostic value of the multi-RNA-based classifier is independent of clinical feature was assessed by multivariate Cox regression model. More importantly, to investigate the performance of the prognostic classifier and clinical features in predicting CRC patient outcome, the area under the curve (AUC) of the receiver-operator characteristic (ROC) was calculated and compared.

We used R software version 3.3.3 and the “TimeROC” package to do the time-dependent ROC curve analysis. Moreover, “glmnet” package was used to do the LASSO Cox regression model analysis. Nomogram plots were done with the rms package. All the other statistical tests were done with R software version 3.3.3 and corresponding fundamental package.
